# Enterovesical Fistulae: Aetiology, Imaging, and Management

**DOI:** 10.1155/2013/617967

**Published:** 2013-11-21

**Authors:** Tomasz Golabek, Anna Szymanska, Tomasz Szopinski, Jakub Bukowczan, Mariusz Furmanek, Jan Powroznik, Piotr Chlosta

**Affiliations:** ^1^Department of Urology, Collegium Medicum of the Jagiellonian University, Ulica Grzegorzecka 18, 31-531 Cracow, Poland; ^2^Department of Interventional Radiology and Neuroradiology, Medical University of Lublin, Ulica Jaczewskiego 8, 20-954 Lublin, Poland; ^3^Department of Endocrinology and Diabetes, Royal Victoria Infirmary, Queen Victoria Road, Newcastle upon Tyne NE1 4LP, UK; ^4^Department of Radiology, Central Clinical Hospital Ministry of Interior in Warsaw, ul. Wolowska 137, 02-507 Warsaw, Poland; ^5^The 1st Department of Urology, Postgraduate Medical Education Centre, European Health Centre in Otwock, ul. Borowa 14/18, 05-400 Otwock, Poland

## Abstract

*Background and Study Objectives*. Enterovesical fistula (EVF) is a devastating complication of a variety of inflammatory and neoplastic diseases. Radiological imaging plays a vital role in the diagnosis of EVF and is indispensable to gastroenterologists and surgeons for choosing the correct therapeutic option. This paper provides an overview of the diagnosis of enterovesical fistulae. The treatment of fistulae is also briefly discussed. *Material and Methods*. We performed a literature review by searching the Medline database for articles published from its inception until September 2013 based on clinical relevance. Electronic searches were limited to the keywords: “enterovesical fistula,” “colovesical fistula” (CVF), “pelvic fistula”, and “urinary fistula”. *Results*. EVF is a rare pathology. Diverticulitis is the commonest aetiology. Over two-thirds of affected patients describe pathognomonic features of pneumaturia, fecaluria, and recurrent urinary tract infections. Computed tomography is the modality of choice for the diagnosis of enterovesical fistulae as not only does it detect a fistula, but it also provides information about the surrounding anatomical structures. *Conclusions*. In the vast majority of cases, this condition is diagnosed because of unremitting urinary symptoms after gastroenterologist follow-up procedures for a diverticulitis or bowel inflammatory disease. Computed tomography is the most sensitive test for enterovesical fistula.

## 1. Introduction

Enterovesical fistula (EVF) represents an abnormal communication between the intestine and the bladder. Although EVF are uncommon, they cause significant morbidity and may markedly affect patient's quality of life. Enterovesical fistulae most frequently occur as a consequence of advanced-stage disease or due to traumatic or iatrogenic injuries. The diagnosis of EVF can be challenging and is often delayed for several months after symptoms begin. Radiological imaging plays a vital role in establishing the site, course, and complexity of fistulae and in identifying an aetiological factor. This paper describes the imaging appearances of enterovesical fistulae and the option for their management.

## 2. Material and Methods

A comprehensive search strategy was applied for Medline/PubMed electronic database from its inception until September 2013. We selected all human research articles published in English, not classified as case report, editorial, comment, letter, or news. The search strategy included the following terms: “enterovesical fistula,” “colovesical fistula”, “pelvic fistula” and “urinary fistula”.

## 3. Results and Discussion

We found a total of 274 papers about urinary tract fistulae and a total of 75 articles specifically related to EVF. Among these, 70 were original articles and 5 were reviews.

### 3.1. Aetiology of Enterovesical Fistulae

It is estimated that enterovesical fistulae account for 1 in every 3,000 surgical hospital admissions [1]. EVF most frequently occur in a setting of inflammatory bowel disease. Diverticulitis is the commonest aetiology accounting for approximately 65–79% of cases, which are almost exclusively colovesical [2–5]. The relative risk for developing enterovesical fistula in the presence of diverticular disease is between 1 and 4% [4, 6]. The underlying mechanism of it is a direct extension of ruptured diverticulum or erosion of a peridiverticular abscess, into the bladder, and a phlegmon and abscess are the risk factors for subsequent fistula formation [4, 5, 7]. The second most common cause of EVF is cancer (10–20% of cases), followed by Crohn's disease (5–7%) [4, 8, 9]. While only approximately 2% of patients with Crohn's disease develop EVF, ileovesical fistula remains the most common type [8, 10]. Regional enteritis, secondary to the transmural inflammation characteristic of Crohn's colitis, may result in adherence to the bladder with subsequent erosion into the organ and further fistula formation [8, 9]. The mean duration of Crohn's disease at the time of onset of EVF-related symptoms is 10 years and an average patient's age is 30 [9]. Less-common inflammatory causes of EVF include Meckel's diverticulum, genitourinary coccidioidomycosis, pelvic actinomycosis, and appendicitis [11–14]. Advanced-stage colon and bladder malignancies account for up to one-fifth of all cases, with the latter being extremely rare [4, 15]. Other urogenital malignancies, as well as lymphoma, cause EVF only occasionally [16, 17]. The iatrogenic aetiology of enterovesical fistulae may occur as a consequence of general surgical procedures (particularly for colorectal cancer, diverticulitis, or inflammatory bowel disease), as well as vascular and urological interventions [18, 19]. Fistulae may also develop as a complication of both chemo- and radiation therapy. External beam radiation or brachytherapy to the bowel in the treatment field can precipitate fistula formation by inducing progressive endarteritis obliterans, which subsequently may result in necrosis and breakdown of mucosal surfaces [20]. Radiation-associated fistulae usually develop years after radiation therapy for gynaecological or urological malignancies [20, 21]. Enterovesical fistulae, secondary to cytotoxic therapy, are extremely rare and have been previously reported in a patient undergoing chemotherapy for non-Hodgkin's lymphoma [22]. Other uncommon causes of EVF include penetrating abdominal or pelvic injuries and foreign bodies in the bowel and peritoneum [5, 23].

### 3.2. Types of Enterovesical Fistulae

Classification of enterovesical fistulae is based on the bowel segment involved. All EVF can be divided into the following 4 primary categories: (i) colovesical, (ii) rectovesical (including rectourethral), (iii) ileovesical, and (iv) appendicovesical fistulae. While colovesical fistula is the most common form of vesicointestinal fistula and is most frequently located between the sigmoid colon and the dome of the bladder, rectovesical fistulae are observed in the postoperative setting (i.e., after prostatectomy) [24]. A key consideration in determining optimal management of EVF is not only the termination point of the fistula tract but also the complexity of the fistula itself. Simple enterovesical fistulae are usually small and single and occur in nonradiated tissue. Complex EVF are larger, have multiple tracts, often develop in a previously irradiated tissue, and are commonly accompanied by a pelvic abscess or a colonic obstruction [2, 21].

### 3.3. Clinical Manifestations and Diagnosis

Symptoms of vesicoenteric fistulae may originate from both the urinary and the gastrointestinal tracts. However, patients with EVF usually present with lower urinary tract symptoms, which include pneumaturia (the most common symptom present in 50–70% of cases), fecaluria (reported in up to 51%), frequency, urgency, suprapubic pain, recurrent urinary tract infections (UTIs), and haematuria [5, 8, 21, 25]. Over 75% of affected patients describe pathognomonic features of pneumaturia, fecaluria, and recurrent UTIs due to *Escherichia coli*, coliform bacteria, mixed growth, or enterococci [4, 5, 21]. The hallmark of enterovesical fistulae is Gouverneur's syndrome characterised by suprapubic pain, frequency, dysuria and tenesmus [26]. Physical signs include malodorous urine and debris in the urine, as well as less commonly reported fever. Additionally, symptoms of an underlying disease causing the fistula may be present. In patients with fistulating Crohn's disease, abdominal pain, abdominal mass, and abscess are more common [27].

### 3.4. Diagnostic Algorithm

The diagnosis of an enterovesical fistula poses a significant challenge as there is no consensus on any clear gold standard for EVF workup. A review of the literature showed that enterovesical fistulae are most commonly diagnosed based on clinical evidence. Nevertheless, diagnostic verification of EVF is necessary not only to establish the presence of a fistula but also to exclude stricture of the bowel and presence of abscess and to evaluate the anatomical region of involved intestine to guide the subsequent surgery [28]. Although cystoscopy, with the highest yield in identifying a potential lesion, is an essential component of the entire investigation process, its findings are usually nonspecific and include erythema, oedema, and congestion. Endoscopic evaluation of the urinary bladder fails to identify EVF in 54–65% of cases [4, 25, 28]. Colonoscopy is not particularly valuable in detecting fistulae. A detection rate for EVF can be as low as 8.5% and does not usually exceed 55% [25, 29, 30]. However, as 10%–15% of colovesical fistulae are secondary to neoplasms, endoscopic examination of the large bowel should be an integral part of CVF workup. It is helpful in determining the nature of the bowel pathology responsible for the fistula formation [4, 5, 25].

The poppy seed test involves oral intake of 50 mg of poppy seeds mixed in beverage or yoghurt. Since seeds remain largely undigested through the gastrointestinal tract, they may appear in urine within 48 hours following intake which is considered a positive confirmatory test for enterovesical fistula. Kwon et al. compared the accuracy of the poppy seed test with CT scanning and nuclear cystography in 20 patients with surgically confirmed fistulae. The poppy seed test yielded a 100% detection rate, whereas CT scanning and nuclear cystography yielded rates of 70% and 80%, respectively. The poppy seeds test is inexpensive and easy to perform; however, it provides little detail regarding the location and type of fistula present [29].

**Figure 1:**
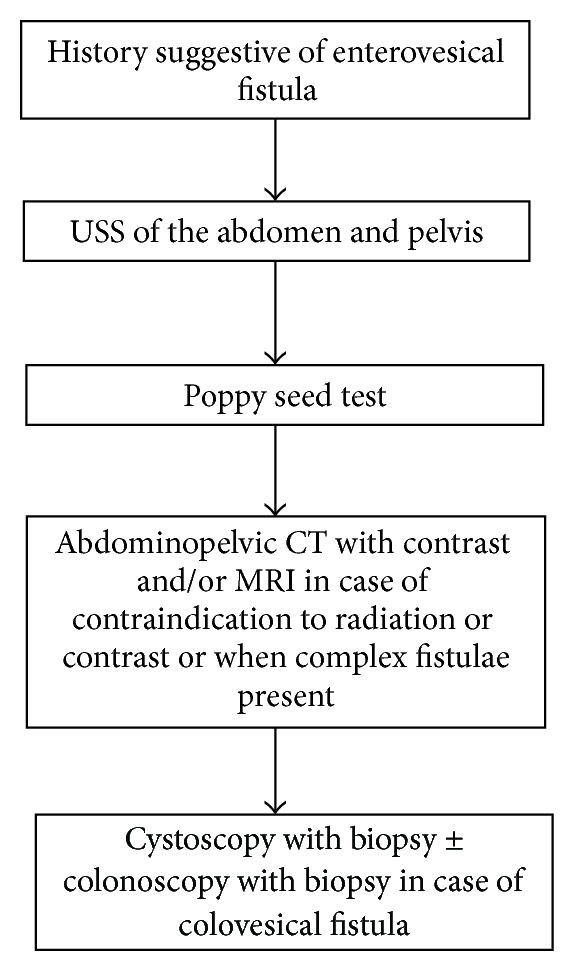
Diagnostic imaging and procedures algorithm for enterovesical fistulae.

The proposed algorithm for diagnosis of enterovesical fistula is presented in Figure 1.

## 4. Imaging Techniques and Appearances

### 4.1. Ultrasonographic Examination

Ultrasonography (USS) may be useful in the diagnosis of colovesical fistulae. In some instances, the fistula is easily identified, with no additional manoeuvers needed [31]. Its detection rate in small series reached 100% [32]. The yield of the transabdominal ultrasonographic examination for suspected fistula can be enhanced by the use of abdominal compression, which reveals an echogenic “beak sign” connecting the peristaltic bowel lumen and the urinary bladder [33]. The identification of the ureteric orifices with their associated urinary jets and the use of lower abdominal compression are essential components of this technique.

**Figure 2:**
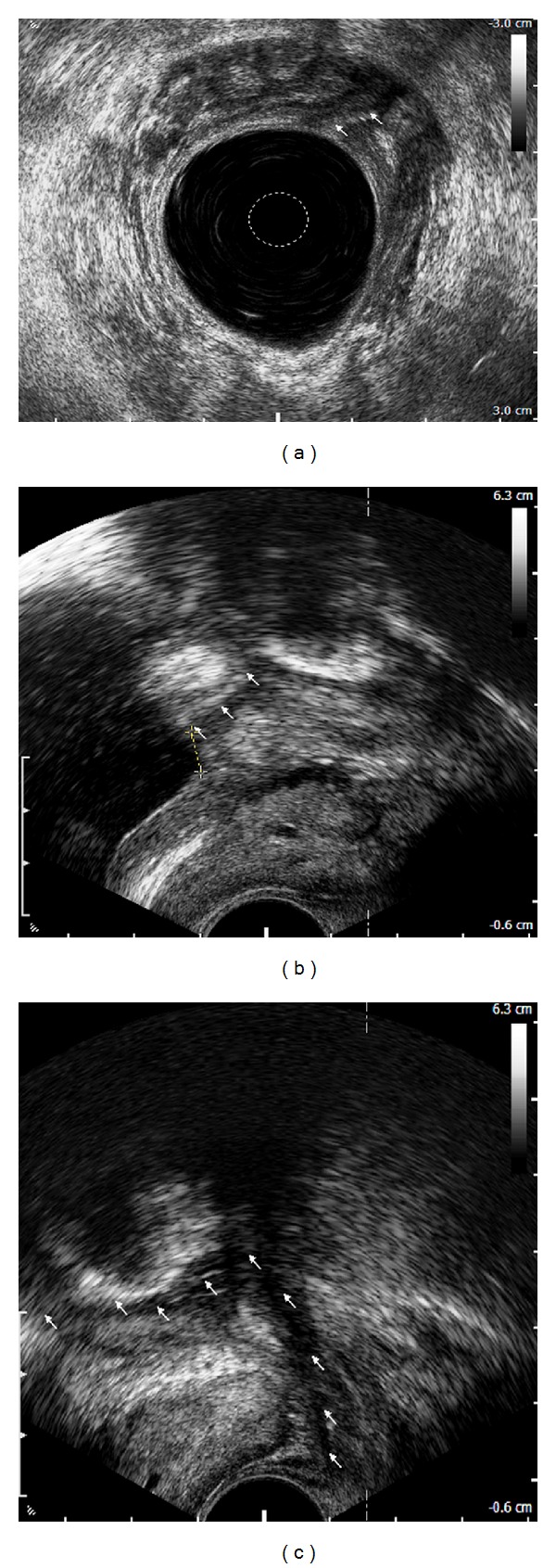
Rectovesical fistula: anorectal endosonographic view of a fistulous orifice in the urinary bladder (arrows) (a), transrectal ultrasonographic view of a fistulous orifice (arrows) located 6 mm from the internal outlet of the bladder (crosses) (b), and transrectal ultrasonographic view of a fistulous tract adjacent to the left lobe of the prostate (arrows) (c).

Anorectal, transrectal, and transvaginal ultrasonography can help to identify a fistulous tract, as well as its relation to the adjacent anatomical structures (Figures 2(a), 2(b), and 2(c)) [34, 35].

### 4.2. Computed Tomography Examination

**Figure 3:**
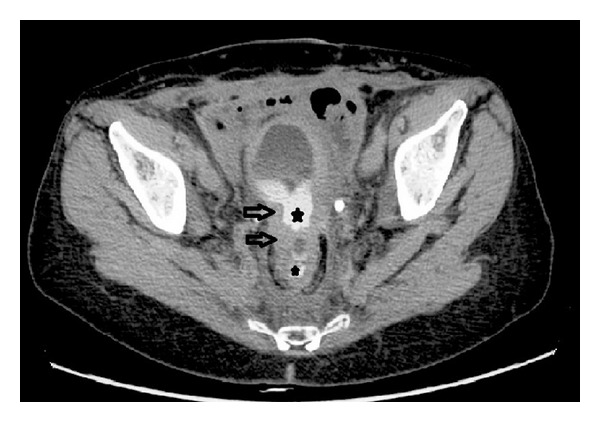
Colovesical fistula: axial image in the delayed phase of CT urogram demonstrates bladder and rectal wall thickening (arrows) with contrast present in both (∗).

**Figure 4:**
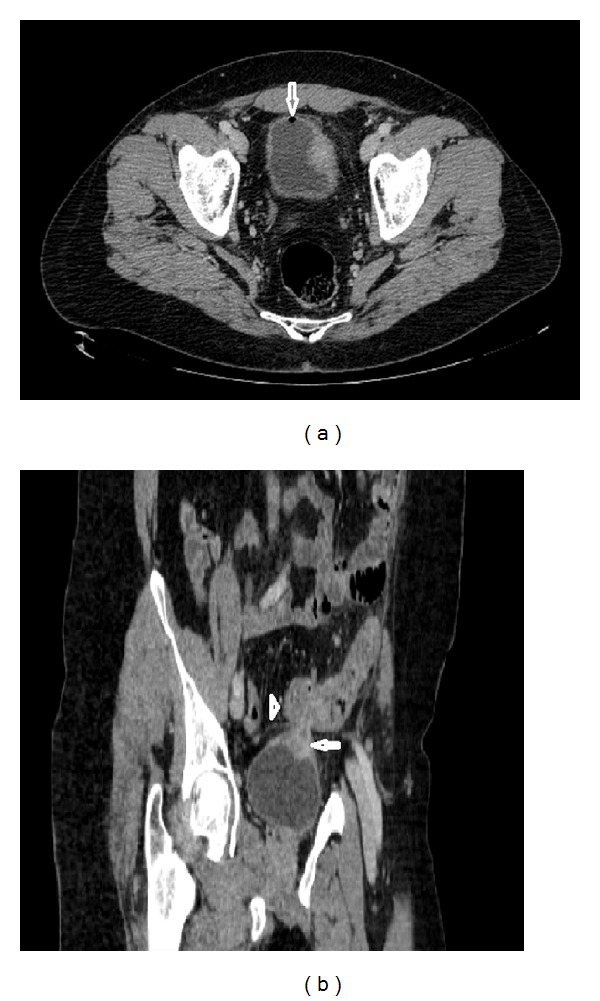
Colovesical fistula: axial image of contrast enhanced CT of the abdomen and pelvis demonstrates air in the bladder (arrow) and thickened left bladder wall (a); sagittal image shows bladder wall thickening (arrow) adjacent to a loop of thickened sigmoid colon (arrow head) (b).

Computed tomography (CT) is the modality of choice for the diagnosis of enterovesical fistulae due to its high sensitivity for the detection of EVF, but more importantly it provides essential additional information about the adjacent anatomical structures [5, 25, 36]. Moreover, the underlying pathology of colovesical fistulae is, in the majority of cases, an extraluminal disease process, and CT scanning is an optimal modality to detect pericolic complications of the diverticular disease [2–5, 7]. The diagnostic accuracy of computed tomography for detecting colovesical fistulae is up to 90–100% [5, 36–38]. CT scanning should be performed following oral administration of contrast but prior to intravenous administration of contrast, in order to permit detection of Gastrografin or other diluted iodinated contrast agents within the bladder. The findings on CT, which are suggestive of enterovesical fistula include (i) air in the bladder (in the absence of previous lower urinary tract instrumentation), (ii) oral contrast medium in the bladder on nonintravenous contrast enhanced scans, (iii) presence of colonic diverticula, and (iv) bladder wall thickening adjacent to a loop of thickened intestine (Figures 3, 4(a), and 4(b)) [4, 5, 37, 39]. The pathognomonic finding of air within the urinary bladder contributes to the high diagnostic accuracy of CT in detecting EVF; however, false positives may occur following recent lower urinary tract instrumentation or due to active urinary tract infection with a gas-forming organism.

Compared with conventional axial CT imaging, 3-dimensional CT provides better visualisation of the anatomical relationship of the bladder and EVF to adjacent structures [40, 41]. Majority of modern CT scanners can acquire a raw data volume enabling almost immediate three planar reconstruction without additional cost.

### 4.3. Magnetic Resonance Imaging

Although computed tomography is the modality of choice in evaluation of colovesical fistulae, the actual fistulous tract is identified on CT only occasionally [36, 37]. Magnetic resonance imaging (MRI) has excellent intrinsic soft tissue resolution together with its multiplanar imagining capability. Moreover, MRI allows accurate depiction of fistulous tract without the necessity of direct opacification required in CT scanning. Its use in colovesical fistulae is well established and its sensitivity and specificity reach up to 100% [25, 42–44]. The appearance of a fistula on MRI depends whether it is filled with fluid, air, or a combination of both. Therefore, the use of combined sequences is ideal. T1-weighted images delineate the extension of the fistula relative to sphincters and adjacent hollow viscera and show inflammatory changes in fat planes. On T2-weighted images, the fistula typically produces a high-signal-intensity, fluid-filled communication, whereas the air-filled fistulous tract is seen as a low signal intensity, regardless of the pulse sequence used [43, 44]. In cases of fistulae due to diverticulitis, abscess (containing high-signal fluid on T2-weighted images) is commonly seen lying between the inferior wall of the sigmoid colon and the superior bladder wall (which is thickened and inflamed) [42].

Use of intravenous gadolinium enhancement significantly improves the detection of bladder fistulae. Early postgadolinium T1-weighted images show enhancement of tract walls and signal void fluid centrally [44]. Both axial and sagittal planes are useful for the detection of enterovesical fistulae [42–44]. Use of short tau inversion-recovery (STIR) images has not been established in the literature yet. Although MRI allows for a precise delineation of fistulous tracts, its high cost and the common lack of MRI access within the emergency room limit its use to more complex elective cases.

### 4.4. Radiographic Examinations

A plain abdominal X-ray is not generally helpful, although when taken with the patient standing may show an air-fluid level within the bladder. Similarly, intravenous urography fails to demonstrate the fistula, unless the patient has severe outlet obstruction [45].

Barium enemas (BE) have a limited role in the diagnosis of enterovesical fistulae due to a low sensitivity of approximately 30% [29, 46]. However, it may be useful in differentiating diverticular disease from colonic cancer as a cause of EVF. Radiographic examination of centrifuged first urine sample obtained immediately after a nondiagnostic BE, called the Bourne test, may significantly enhance the yield of the barium study [46, 47]. Radiodense particles detected in the urine sediment confirm the presence of a fistula [40, 41]. A detection rate for colovesical fistulae can even reach 90% [46]. However, currently its role in an enterovesical fistula workup is marginal since CT and other more advanced studies provide explicit information regarding not only the presence or absence of a fistula but more importantly about its location, complexity, and surrounding anatomical structures. 

Enterovesical fistulae may be evaluated with cystography which may demonstrate contrast outside the bladder; however, it is less likely to demonstrate a fistula [25]. A detection rate for enterovesical fistula ranges between 20% and 30% [29]. The herald sign is a crescentic defect on the upper margin of the bladder and it represents a perivesical abscess. The pathognomonic finding of colovesical fistula is the “beehive” sign caused by the elevation of the bladder wall at the vesical end of the fistulous tract [5, 48]. 

The use of Tc-99 m DTPA as a valuable method in diagnosis of enterovesical fistula has been reported [49, 50]. It is a simple and readily available tool, which provides anatomic as well as functional information about the urinary tract. Moreover, it may demonstrate the presence and location of a fistula indicated by the passing of the radioactive urine from the urinary system into the bowel. The severity of EVF can be determined by assessing the urine flow rate that passes through the fistula [51].

Because of the superiority of CT scanning, as a tool for diagnosis and treatment planning, plain cystography, and radionuclide renography are only occasionally used in the evaluation of enterovesical fistulae. 

Advantages and disadvantages of diagnostic tests and procedures used for detection of enterovesical fistulae are presented in Table 1.

## 5. Management of Enterovesical Fistulae

### 5.1. Conservative Management

Nonoperative treatment of enterovesical fistulae may be an option in nontoxic, minimally symptomatic patients with nonmalignant EVF origin, particularly in those with Crohn's disease. A trial of medical therapy including bowel rest, total parenteral nutrition, antibiotics, steroids, immunomodulatory drugs, and urethral catheter drainage may be warranted [52]. In patients with colovesical fistulae, conservative management has been reported to be associated with the same disease-specific mortality as with the surgical treatment [28, 53]. However, others have found significantly more deaths related to poor physical condition, progression of malignant disease, and the septic effect of the EVF [30, 54]. Therefore, nonsurgical management of colovesical fistulae is generally reserved for patients unfit for major intervention or with extensive unresectable neoplastic process. In those cases, medical therapy with catheter drainage of the bladder alone or supravesical percutaneous diversion could be beneficial. However, most patients will require a diverting stoma in due course of a disease.

### 5.2. Surgical Repair

Endoscopic, open, and laparoscopic approaches have all been used in surgical treatment of enterovesical fistulae [2, 8, 52, 55–59]. Colonoscopic closure of iatrogenic perforations <1 cm is a valuable option of a minimally invasive treatment. In such cases, repair of the perforation can be achieved using the TriClip device [55]. Endoscopic treatment of enterovesical fistulae due to colorectal cancer is commonly associated with bowel stenosis and requires the use of covered self-expanding metal stents. This technique allows for the application of stents within the stenotic bowel segment, even in the presence of a neoplastic, fragile tissue, without further narrowing of its lumen. However, this method is contraindicated in the management of enterovesical fistula caused by diverticulitis as stent placement is associated with high risk of the colon perforation [60].

Operative management of enterovesical fistulae is mainly dependent on the underlying pathology, site of the bowel lesion, and patient's preoperative status. Both open and laparoscopic approaches have been used for the treatment of enterovesical fistula [2, 8, 57, 58]. The aim of operative management is to resect and reanastomose the offending bowel segment and to close the bladder. The treatment may involve single-stage or multistage procedures [2, 52]. The former involves resection and primary anastomosis without a protective colostomy, whereas, during the latter, resection and primary anastomosis with colostomy and/or Hartmann procedure are performed (two-stage procedure) with later closure of the stoma (three-stage approach). Staged procedures have been advocated in patients with gross faecal contamination and large intervening pelvic abscesses or in those with advanced malignancy or radiation changes [2, 8]. Historically, proximal defunctioning procedures as sole interventions have been recommended in the management of EVF [61]. Although they are associated with low surgical trauma, they are unlikely to result in a fistula tract closure. Moreover, a fistula often recurs following reversal of a colostomy and patients may still remain prone to urinary sepsis [53]. 

Bowel resection with primary anastomosis is advocated in the majority of EVF cases [2, 25]. Successful one-stage resections have been reported in 18% to 92% of EVF cases [54, 62]. Surgical technique involves blunt dissection of the bowel from the bladder, resection of the intestine, and primary anastomosis. As an opening of a fistulous tract in the bladder may not be directly visible, distention of the bladder with methylene blue solution instilled through a catheter may be helpful. The type of bladder repair, whether excision or oversewing, is not of critical importance since small defects do not require closure and may be left to heal spontaneously [63]. Although no strong evidence is available, if technically possible, interposition of the omental flap between the bladder and intestine may be employed. Such maneuver might improve healing process and reduce the fistula recurrence rate due to high vascularity and immunological properties of the omentum [64].

Surgical management of radiation-induced enterovesical fistulae is challenging and in severe cases impossible as no clear planes between the anatomical structures can be identified. Moreover, radiation-induced fistulae are more likely to recur. Hence, in such patients, a proximal defunctioning stoma may be an option as it can improve their quality of life. 

The outcome of enterovesical fistulae management is, in the majority of cases, excellent. Postoperative recurrence of EVF is uncommon in patients with benign and nonradiation-induced fistulae. Persistence of a fistula after presumably definitive treatment may also be related to malignancy, nutritional issues, unrecognised foreign body, or surgical factors.

## 6. Conclusions

Enterovesical fistulae are an uncommon complication of both benign and malignant processes. The diagnosis of EVF may, however, be challenging. With a high index of suspicion for fistula formation in patients presenting with symptoms suggestive of abnormal communication between the intestine and the bladder, appropriate radiological investigation can lead to a significant reduction in morbidity. Recognition of a fistulous tract, delineation of its course, and characterisation of its complexity affect the EVF management. In this respect, cross-sectional imaging with CT and MRI remains an ideal modality option in patients with enterovesical fistulae. Management of EVF is mainly dependent on the underlying pathology, site of the bowel lesion, and patient's preoperative performance status. Surgical one-stage strategy is a preferred option in most of the cases.

## Figures and Tables

**Table 1:** Advantages and disadvantages of diagnostic tests and procedures used for the detection of enterovesical fistulae.

Modality	Advantages	Disadvantages
Cystoscopy	Direct visualisation of the bladder Allows for the biopsy of a lesion	Invasive test Visualises only intraluminal content Success rate of 35%–46%

Colonoscopy	Helps to identify bowel pathology that caused a colovesical fistula	Invasive test Visualises only intraluminal content Success rate of 8.5%–55%

Poppy seed test	Noninvasive Inexpensive Convenient to perform Accuracy of up to 100%	Does not provide information on fistula location and type

Transabdominal ultrasonography	No X-ray exposure Inexpensive and available Success rate of up to 100%	Does not provide more detailed information regarding complexity of a fistula

Abdominopelvic CT	Modality of choice Diagnostic accuracy between 30 and 100% Provides information about the complexity of a fistula and the surrounding anatomical structures	X-ray exposure Expensive Often fails to identify fistulous tract

MRI	No X-ray exposure Helpful in complex cases Success rate of up to 100%	Expensive Limited availability

Barium enema	Useful in differentiating diverticular disease from colonic cancer Low perforation rates (<1%)	X-ray exposure Barium peritonitis Visualises only intraluminal content Detection rate of approximately 30%

Bourne test	Inexpensive Detection rate for colovesical fistulae of up to 90%	Does not provide information on fistula location and type

Cystogram	Easy to perform Available	X-ray exposure Low detection rate Does not provide information on fistula location Not helpful in case of a complex fistula
